# Neighborhood-Level Socioeconomic Deprivation, Rurality, and Long-Term Outcomes of Patients Undergoing Total Joint Arthroplasty: Analysis from a Large, Tertiary Care Hospital

**DOI:** 10.1016/j.mayocpiqo.2022.06.001

**Published:** 2022-07-01

**Authors:** Celia C. Kamath, Thomas J. O’Byrne, David G. Lewallen, Daniel J. Berry, Hilal Maradit Kremers

**Affiliations:** a Department of Quantitative Health Sciences, Mayo Clinic, Rochester, MN; b Department of Orthopedic Surgery, Mayo Clinic, Rochester, MN

**Keywords:** ADL, activities of daily living, HR, hazard ratio, PJI, periprosthetic joint infections, RUCA, rural-urban commuting area, SES, socioeconomic status, TJA, total joint arthroplasty

## Abstract

**Objective:**

To assess the impact of neighborhood-level socioeconomic status factors (area deprivation index [ADI] and rural classification) and their interaction with individual-level socioeconomic status (education-level) on long-term outcomes following total joint arthroplasty (TJA) surgery.

**Patients and Methods:**

This was a cohort study of 46,828 TJA surgeries performed on patients at a tertiary care hospital between January 1, 2000 and December 31, 2019. Cox proportional hazards models were used to examine the association between ADI and rurality and their interaction with individual-level education on the risk of periprosthetic joint infections, revision surgery, and mortality.

**Results:**

At the time of surgery, 2589 (6%) patients lived in the most deprived neighborhoods (ADI quintile >80%) and 10,728 (23%) lived in small isolated rural towns. Patients from the most deprived neighborhoods were more likely to experience revision surgery (hazard ratio, [HR], 1.39; 95% CI, 1.10-1.76) and mortality (HR, 1.24; 95% CI, 1.09-1.42). Patients from small rural towns were also more likely to undergo revision surgery (HR, 1.14; 95% CI, 1.01-1.28). The mortality risk was 13%, 18%, and 24% higher for patients in the 3 highest ADI quintiles than those from the lowest quintile. Education gradient was more notable in the least deprived neighborhoods than in the most deprived neighborhoods.

**Conclusion:**

Neighborhood disadvantage and rurality are negatively associated with the risk of revision surgery and both independently and in interaction with individual-level education with the risk of mortality. There is a need for population-level health interventions to mitigate area-based socioeconomic disadvantages in TJA.

Osteoarthritis is the most prevalent joint disease.^1^ Total joint arthroplasty (TJA) of the hip and knee joints are effective surgical procedures for patients with advanced osteoarthritis. Total joint arthroplasties are, in fact, the most common surgeries with nearly 2 million procedures annually in the United States.^2^ However, despite technological progress and increase in the number of surgeons performing these procedures, disparities based on race and individual socioeconomic status (SES) have persisted over time.^3^, ^4^, ^5^, ^6^

There is growing evidence of geographical- and area-based underpinning of disparities in TJA care,^7^ adding to disparities that stem from individual-level factors. Further, the relationship between area- and individual-based sources of deprivation is poorly understood. The development of effective strategies to combat disparities in access to TJA care and surgical outcomes depends on such exploration. This issue is even more compelling with value-based payment reforms,^8^, ^9^, ^10^, ^11^ in which the objective is to reduce costs and improve outcomes. By failing to take into account the disparities associated with TJA surgical outcomes, we run the risk of penalizing institutions that provide care to the economically vulnerable and underserved patients who underwent TJA, thus encouraging discrimination against these populations.^10^, ^11^, ^12^, ^13^ Exploring the role of these factors and relationships between them would advance the understanding on how social factors become “embodied” in the risk for postsurgery outcomes and enable considerations for appropriate interventions and payment policy adjustments.^14^

The aim of this study was to examine the association between neighborhood-based deprivation measures and their interaction with individual-level SES factors on long-term surgical outcomes in patients who underwent TJA. We specifically examined the association between area deprivation indices and rural classification of patients’ residences, and their interaction with one aspect of individual-level SES (education-level) on long-term surgical outcomes. Education level is considered a close proxy for income and was available in our data, whereas income or insurance status was not. We recognize the heterogeneity of individual-level SES within neighborhoods with different levels of deprivation. Examining the interaction between individual-level SES factors (ie, education) and area-based factors will provide insight into the additive influences, if any, of neighborhood and individual-level SES on outcomes. We also examined the association of nursing home residency on these outcomes. Residents of nursing homes are likely to make and be constrained by choices, different from independent residential dwellers. Our aim was thus to explore the independent effect and interplay between these area-based sources of deprivation and at least 1 individual-level SES factor, education, on long-term surgical morbidity and mortality.

## Patients and Methods

Institutional review board approval was obtained for this study. The study population comprised a retrospective cohort of 33,287 individuals with 46,828 surgeries (24,422 total knee arthroplasty and 22,406 total hip arthroplasty with 18.4% revision surgeries) at a large tertiary care hospital between January 1, 2000 and December 31, 2019 (Table 1). Patient demographic characteristics (age, sex, race, ethnicity, and highest level of education), behavioral factors (body mass index [calculated as the weight in kilograms divided by the height in meters squared] and smoking), surgical characteristics (surgery type and American Society of Anesthesiologists score), and patient-reported measures (basic and instrumental activities of daily living [ADL]) were derived from patients’ electronic health records and the institutional joint registry. Information on highest level of education was extracted from patients’ self-reported information and classified as a binary education variable as high school or less or greater than high school. Comorbidities recorded at the index hospitalization, before actual surgery, or the 2-year time window before hospitalization were identified and classified using the Charlson comorbidity index categories.

**Table 1:** Characteristics of the Study Population by Quintiles of Area Deprivation Index (ADI)

Characteristic	ADI quintile, 1-20	ADI quintile, 21-40	ADI quintile, 41-60	ADI quintile, 61-80	ADI quintile, 81-100	Total	*P* for trend
No (%) of TJA surgeries	6649 (14)	13,939 (30)	14,533 (31)	9118 (19)	2589 (6)	46,828	
Age (y), mean ± SD	66.0 (11.72)	66.1 (12.20)	67.5 (12.60)	67.3 (13.23)	67.3 (13.55)	66.8 (12.56)	<.0001
Women, n (%)	3363 (50.6)	7513(53.9)	8015(55.2)	5223(57.3)	1562(60.3)	25,676(54.8)	<.0001
Non-White race, n (%)	388 (5.8)	562(4.0)	548(3.8)	485(5.3)	201(7.8)	2184(4.7)	<.0001
Hispanic ethnicity, n (%)	48 (0.8)	100(0.8)	98(0.8)	77(1.1)	28(1.4)	351(0.9)	.05
Highest education level, n (%)							<.0001
High school or less	839 (12.7)	3850(27.9)	5352(37.4)	3635(40.7)	1065 (41.7)	14,741 (31.9)	
More than high school	5703 (86.6)	9819(71.3)	8844(61.7)	5199(58.3)	1448 (56.7)	31,013 (67.2)	
BMI, mean (SD), kg/m^2^	29.8 (6.18)	31.1(6.64)	31.3(6.80)	31.5(6.94)	31.4 (7.12)	31.1 (6.73)	<.0001
Smoking, n (%)							<.0001
Never	2638 (55.5)	5284(54.1)	4962(53.1)	2914(52.4)	795(51.0)	16,593(53.5)	
Current	203 (4.3)	565(5.8)	660(7.1)	489(8.8)	172(11.0)	2089(6.7)	
Previous (quit date unknown)	103 (2.2)	216(2.2)	201(2.2)	113(2.0)	35(2.2)	668(2.2)	
Quit >6 wk ago	1739 (36.6)	3546(36.3)	3337(35.7)	1931(34.7)	521(33.4)	11,074(35.7)	
Quit <6 wk ago	19 (0.4)	56 (0.6)	71 (0.8)	45 (0.8)	15 (1.0)	206 (0.7)	
Instrumental ADL limitation, n (%)	897 (14.2)	2212 (16.8)	2492 (18.1)	1833 (21.5)	653 (26.9)	8087 (18.3)	<.0001
Basic ADL limitation, n (%)	4682 (72.4)	9612 (71.1)	10,027 (71.7)	6298 (72.4)	1839 (74.6)	32,458 (71.9)	.004
Joint, n (%)							.001
THA	3204 (48.2)	6516 (46.7)	6912 (47.6)	4489 (49.2)	1284 (49.6)	22,405 (47.8)	
TKA	3444 (51.8)	7423 (53.3)	7621 (52.4)	4629 (50.8)	1305 (50.4)	24,422 (52.2)	
Surgery type, n (%)							<.0001
Primary	5545 (83.4)	11,487 (82.4)	11,951 (82.2)	7227 (79.3)	1990 (76.9)	38,200 (81.6)	
Revision	1103 (16.6)	2452 (17.6)	2582 (17.8)	1891 (20.7)	599 (23.1)	8627 (18.4)	
ASA score, n (%)							<.0001
I-II	4131 (62.1)	7930 (56.9)	7560 (52.0)	4474 (49.1)	1227 (47.4)	25,322 (54.1)	
III-V	1809 (27.2)	4438 (31.8)	4946 (34.0)	3235 (35.5)	934 (36.1)	15,362 (32.8)	
Comorbidities, n (%)	247 (4.5)	693 (6.2)	891(7.6)	508 (6.8)	166 (7.9)	2505 (6.6)	<.0001
Myocardial infarction	356 (6.5)	1013 (9.1)	1252 (10.7)	811 (10.9)	234 (11.1%)	3666 (9.7%)	<.0001
Congestive heart failure	962 (17.6)	2133 (19.1)	2395 (20.5)	1399 (18.8)	418 (19.9)	7307 (19.3)	.0001
Peripheral vascular disease	544 (9.9)	1189 (10.6)	1433 (12.3)	865 (11.6)	218 (10.4)	4249 (11.2)	<.0001
Cerebrovascular disease	137 (2.5)	382 (3.4)	457 (3.9)	288 (3.9)	86 (4.1)	1350 (3.6)	<.0001
Dementia	1031 (18.8)	2594 (23.2)	2586 (22.1)	1550 (20.8)	527 (25.0)	8288 (21.9)	<.0001
Chronic pulmonary disease	297 (5.4)	684 (6.1)	829 (7.1)	494 (6.6)	165 (7.8)	2469 (6.5)	<.0001
Ulcer	446 (8.2)	999 (8.9)	985 (8.4)	585 (7.9)	195 (9.3)	3210 (8.5)	.06
Mild liver disease	665 (12.2)	1736 (15.5)	1994 (17.0)	1302 (17.5)	410 (19.5)	6107 (16.1)	<.0001
Diabetes							
Diabetes with end-organ damage	181 (3.3)	548 (4.9)	632 (5.4)	397 (5.3)	116 (5.5)	1874 (4.9)	<.0001
Hemiplegia	77 (1.4)	175 (1.6)	203 (1.7)	160 (2.1)	36 (1.7)	651 (1.7)	.01
Renal disease	403 (7.4)	1003 (9.0)	1252 (10.7)	773 (10.4)	231 (11.0)	3662 (9.7)	<.0001
Moderate or severe liver disease	63 (1.2)	176 (1.6)	178 (1.5)	99 (1.3)	37 (1.8)	553 (1.5)	.13
Metastatic solid tumor	211 (3.9)	470 (4.2)	572 (4.9)	314 (4.2)	98 (4.7)	1665 (4.4)	.01
Rheumatologic disease	385 (7.0)	919 (8.2)	1039 (8.9)	651 (8.7)	193 (9.2)	3187 (8.4)	.0005
Cancer	889 (16.2)	1949 (17.4)	2130 (18.2)	1291 (17.3)	381 (18.1)	6640 (17.5)	0.03
RUCA categorization, n (%)							<.0001
Urban	6432 (96.8)	10442 (74.9)	7573 (52.1)	3001 (32.9)	1146 (44.3)	28594 (61.1)	
Large rural town	120 (1.8)	2150 (15.4)	2798 (19.3)	1888 (20.7)	549 (21.2)	7505 (16.0)	
Small/isolated rural town	96 (1.4)	1347 (9.7)	4162 (28.6)	4229 (46.4)	894 (34.5)	10728 (22.9)	
Nursing home resident, n (%)	22 (0.3)	240 (1.7)	301 (2.1)	285 (3.1)	71 (2.7)	919 (2.0)	<.0001

ADI, area deprivation index; ADL, activities of daily living; ASA, American Society of Anesthesiologists; BMI, body mass index; RUCA, Rural-Urban Commuting Area; THA, total hip arthroplasty; TJA, total joint arthroplasty; TKA, total knee arthroplasty.

Number (%) of surgeries with missing data were as follows: ethnicity, 7263 (15%); education, 1073 (2%); BMI, 541 (1%); smoking, 16,198 (35%); instrumental ADL, 2591 (6%); basic ADL, 1670 (4%); and ASA, 10,493 (22%).

The 3 outcomes of interest were periprosthetic joint infections (PJIs), revision or rerevision surgery, and mortality. All 3 outcomes are important long-term outcomes affected by access to timely care, in which both ADI (in terms of affordability and resources) and rurality (in terms of geographic access) are likely to influence timely access to care. Long-term follow-up of all patients who underwent TJA in our institution were performed by the surgeons and/or trained registry personnel using standard operating procedures and predefined data collection forms. Patients were followed-up twice during the first year of surgery, then in years 2 and 5 and thereafter at 5-year intervals to collect information on all TJA outcomes. If in-person follow-up was not possible, patients were contacted by letter and/or telephone and requested to complete a standardized form that included information for all of these outcomes.

The 2 exposure variables were the patients’ neighborhood area deprivation index (ADI), signifying socioeconomic means, and degree of rurality, signifying ease of health care access. Each patient’s address geolocation was calculated by linking addresses to the TIGER/Line address range shapefile provided by the US Census. Non-US patients were excluded. Area deprivation index were calculated based on the 2015 American Census Survey results at the census block group level, the smallest geographic unit for which the US Census Bureau publishes sample data.^15^ Patients were assigned a rurality index, defined by census-tract level 2010 Rural-Urban Commuting Area (RUCA) codes. We used the census geocoder, an address look-up tool, publicly available to derive census block groups for each patients’ address.^16^ The 2010 census data was used to geocode each address. The 2015 American Community Survey^17^ was used in conjunction with the University of Wisconsin’s Neighborhood Atlas data^7^ to derive an ADI score for each address. The ADI was divided into quintiles for analysis, in which census blocks in lower quintiles represented areas of lower deprivation. Rurality was assigned using patient’s geocoded data transformed into individual Federal Information Processing System codes that were then used to assign a rurality code (urban, large rural, and small isolate rural) for each address with RUCA codes available through the US Department of Agriculture.^18^ Foreign patients and patient addresses that could not be matched to a census block group or could not be assigned a RUCA code or assisted living-nursing home address were excluded from our analysis.

We also noted if the patient’s address was flagged as that of a nursing home or assisted living facility. In addition, we cross-referenced all other addresses with Google maps to check if the address was that of an assisted living-nursing home. We chose to create this separate category because assisted living-nursing homes as a geographic entity are residential care facilities that are different from the other neighborhood-based variables, ADI, and rurality. We assumed that assisted living-nursing homes were fairly stable entities over the 2 decades relevant to our research.

We first examined univariate associations between ADI quintile, rural classification, and nursing home status with PJI, revision, and mortality outcomes. We then used multivariable Cox proportional hazard models for each outcome, adjusting for age, sex, race, education, body mass index >30 kg/m^2^, smoking status, ADL limitations, American Society of Anesthesiologists score, and comorbidities. We examined 2-way interactions between ADI and education. Cox models were appropriate for this study because we are examining long-term outcomes that occur several years after surgery, follow-up varies across patients and the censoring times were different for each outcome.

## Results

At the time of surgery, most patients lived in the mid ADI quintiles (ADI quintile 21-40 and 41-60) with only 2589 (6%) patients living in the most deprived neighborhoods (ADI quintile >80%) and 10,728 (23%) in small isolated rural towns (Table 1). Patients from the least deprived neighborhoods (ADI quintile, 1-20) were more likely to live in an urban area, whereas those from the higher quintiles were more likely to live in a rural area. Patients from higher ADI quintiles (more deprived) were more likely to be women, of non-White race, Hispanic ethnicity, obese, current smokers, and with an education of high school or less. They also had a significantly higher prevalence of several comorbidities. During a mean follow-up of 5.8 years, 1022 (2%) surgeries were complicated with PJI, 2647 (6%) underwent at least 1 revision surgery, and 10,425 (22%) patients died.

### Periprosthetic Joint Infections

In univariate analyses, education level, ADI, rurality, and nursing home status were all associated with a higher risk of PJI. Patients with an education level of high school or less were more likely to experience PJI (HR, 1.23; 95% CI, 1.08-1.39). Compared with patients from the least deprived neighborhoods, patients from the most deprived neighborhoods (ADI quintile >80%) had a 60% higher likelihood of PJI (HR, 1.60; 95% CI, 1.12-2.13) with a significant trend with worsening ADI (*P* value for trend <.01). Patients from both large (HR, 1.31; 95% CI, 1.12-1.54) and small/isolated rural towns (HR, 1.16; 95% CI, 1.00-1.35) and nursing home residents (HR, 1.59; 95% CI, 1.07-2.38) were also more likely to experience PJI. However, in adjusted analyses, none of the neighborhood deprivation indices were associated with the risk of PJI (Table 2). There was no interaction between individual-level education and ADI, rurality, or nursing home residency on the risk of PJI (*P* values of .28, .29, and .55, respectively).

**Table 2:** Association of Area Deprivation Index With TJA Outcomes

	Hazard ratio (95% CI)					
	Periprosthetic joint infection (PJI)		Revision surgery		Mortality	
	Univariate	Multivariable^a^	Univariate	Multivariable^a^	Univariate	Multivariable^a^
ADI quintile						
Fifth quintile (81-100)	1.60 (1.20-2.13)	1.29 (0.89-1.86)	1.52 (1.27-1.83)	1.39 (1.10-1.76)	1.74 (1.58-1.91)	1.24 (1.09-1.42)
Fourth quintile (61-80)	1.27 (1.01-1.58)	0.95 (0.71-1.27)	1.14 (0.99-1.31)	1.07 (0.89-1.28)	1.62 (1.50-1.74)	1.18 (1.06-1.30)
Third quintile (41-60)	1.19 (0.96-1.46)	0.98 (0.75-1.28)	1.13 (0.99-1.29)	1.07 (0.91-1.27)	1.47 (1.37-1.57)	1.13 (1.02-1.24)
Second quintile (21-40)	1.16 (0.93-1.43)	1.01 (0.77-1.32)	1.26 (1.11-1.44)	1.18 (1.00-1.40)	1.28 (1.19-1.38)	1.09 (0.99-1.20)
First quintile (1-20)	Ref	Ref	Ref	Ref	Ref	Ref
Rurality						
Large rural town	1.31 (1.12-1.54)	1.14 (0.92-1.41)	1.10 (0.99-1.22)	1.04 (0.91-1.20)	1.10 (1.05-1.16)	1.05 (0.97-1.13)
Small rural town	1.16 (1.00-1.35)	1.10 (0.91-1.33)	1.09 (1.00-1.20)	1.14 (1.01-1.28)	1.12 (1.07-1.17)	1.04 (0.98-1.11)
Urban	Ref	Ref	Ref	Ref	Ref	Ref
Nursing home residency						
Nursing home resident	1.59 (1.07-2.38)	1.31 (0.74-2.32)	1.18 (0.87-1.59)	1.25 (0.82-1.88)	3.84 (3.53-4.18)	1.45 (1.29-1.63)
Not nursing home	Ref	Ref	Ref	Ref	Ref	Ref

ADL, activities of daily living; BMI, body mass index; TJA, total joint arthroplasty.

a Adjusted for age, sex, race, education, BMI > 30 kg/m^2^, smoking status, ADL limitations, American Society of Anesthesiologists score, and comorbidities.

### Revisions

In univariate analyses, we observed statistically significant associations between ADI, and rurality with the risk of revision surgery, but no association of education level or nursing home residency status with this outcome. Compared with patients from the least deprived neighborhoods, those from the most deprived neighborhoods were more likely to undergo revision surgery (HR, 1.52; 95% CI, 1.27-1.83) as were residents of small/isolated rural towns (HR, 1.09; 95% CI, 1.00-1.20). These associations persisted in adjusted analysis (Table 2). We did not observe any interaction between area-level deprivation indices and individual-level education on the risk of revisions (*P*=.99).

### Mortality

In univariate analyses, all neighborhood-based deprivation measures were statistically significant associated with mortality. In adjusted analysis, the risk of mortality, although attenuated relative to univariate associations, increased with increasing ADI. Compared with the least deprived neighborhoods, mortality was 15%-30% higher for those living in the third (HR, 1.13; 95% CI, 1.02-1.24), fourth (HR, 1.18; 95% CI, 1.06-1.30), and fifth (HR, 1.24; 95% CI, 1.09-1.42) quintiles. Mortality was also significantly higher for those living in nursing homes (HR, 1.45; 95% CI, 1.29-1.63) and those with lower education level (HR, 1.25; 95% CI, 1.19-1.32).

We observed a significant interaction between ADI and individual-level education (*P*<.0001) on mortality risk (Figure). Regardless of ADI, patients with higher education had lower risk for mortality than lesser educated patients. In the least deprived neighborhoods (ADI quintile, 1-20), mortality risk was 32% higher for those with education high school or less (HR, 1.32; 95% CI, 1.09-1.60) vs those with education level more than high school (reference group). Mortality risk progressively increased with increased area deprivation for those with less than high school education to as much as 70% higher for the most deprived ADI quintile (ADI quintile, 81-100) compared with reference group (patients with education level more than high school in the least deprived neighborhoods).

**Figure:**
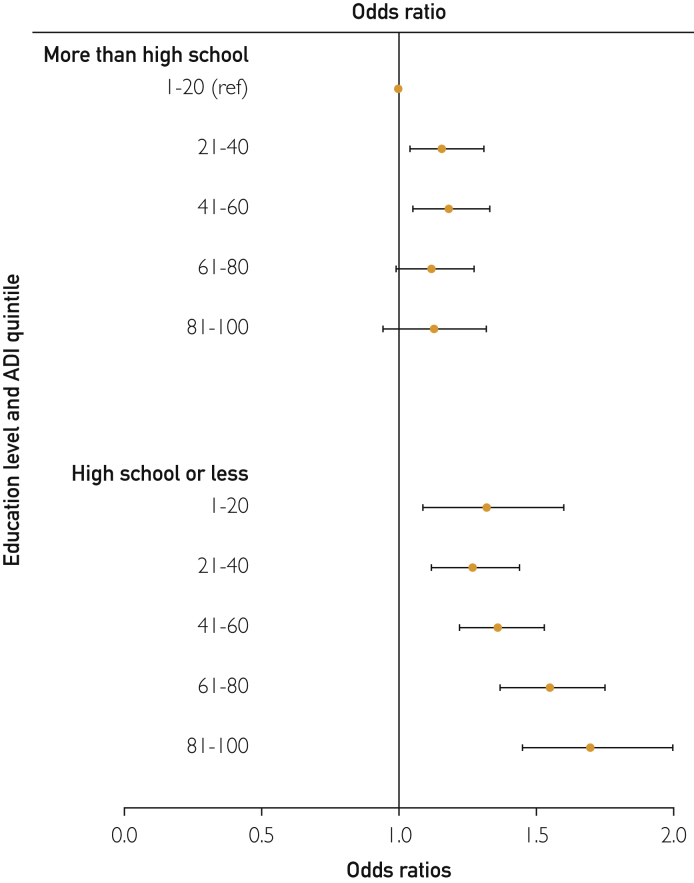
Interaction between area deprivation index (ADI) and Education level on mortality risk following total joint arthroplasty. Education level: more than high school and less than high school. ADI quintiles: 1-20 lowest deprivation quintile and 81-100 highest deprivation quintile.

## Discussion

In this large cohort of patients who underwent TJA surgery at a large tertiary care center, area deprivation indices, ie, ADI, rurality, and nursing home residency, were associated with postsurgical infections, revision surgery, and mortality. Each of these indices indicate different aspects of deprivation: the ADI measures neighborhood-level socioeconomic deprivation, rurality indicates geographic access to health care, and nursing home residency indicates an institutional setting where caregiving is not administered by family members. We hypothesized that all 3 indices would be associated with TJA outcomes. However, after adjusting for patient characteristics and comorbidities, ADI and nursing home status were associated only with the risk of mortality and the highest level of rurality (small rural towns) with the risk of revision surgery. These findings indicate that, irrespective of neighborhood-level deprivation or geographic disadvantage, patients who had access to TJA surgery at a high-volume tertiary center with subspecialty TJA care have relatively good long-term outcomes.

This is one of the few studies examining the role of neighborhood-based deprivation factors on TJA outcomes of infections, revisions, and mortality. Most studies to date examined zipcode-level income as a measure of SES,^3^ but because zipcodes cover a relatively large population, they are more biased than measures from smaller geographic units such as the ADI at the census block level.^19^^,^^20^ Nevertheless, our findings are broadly comparable with findings from other outcome disparities research in patients who underwent TJA. For example, disparities in WOMAC scores between Black and White patients were more evident in areas of higher neighborhood-level deprivation.^21^ Similarly, zipcode-level household income was found to be associated with the length of stay and readmissions.^22^^,^^23^ Our findings on the association of individual-level education on mortality echo the findings of studies in the general population and in patients who underwent TJA,^3^^,^^24^^,^^25^ although we did not find any associations with risk of infections and revision surgery. A potential reason for our observation of weaker associations of ADI with revision surgery and infection outcomes in in patients who underwent TJA is selection bias.^26^ Even though socioeconomic gradient in access to TJA is very strong,^6^^,^^27^, ^28^, ^29^ for disadvantaged patients (based on residency, rurality, or nursing home status) who manage to access TJA care at a high-volume specialty care hospital, the association between ADI and TJA outcomes is attenuated.

Individual-level SES measured indirectly by the educational level was also associated with mortality. In addition, individual-level and neighborhood-level socioeconomic deprivation interacted, moderating the effect of the latter on mortality: patients with lower education residing in areas of higher socioeconomic deprivation were less likely to suffer mortality than patients with lower education living in areas with the least economic deprivation. However, in patients with low SES, mortality rates linearly increased as area deprivation increased. These findings are similar general population trends.^30^^,^^31^

Notably, the rurality of patients’ residences was not associated with TJA outcomes, with a single exception; residents of both small and large rural towns were more likely to have revision surgery compared with residents from urban settings. This finding is similar to previous studies concerning higher rates for TJA surgery in rural areas relative to urban settings.^28^^,^^32^ It has been suggested that this may be related to geographic access to care, rather than deprivation. Revision surgery can be used as a substitute for ongoing conservative care for patients living in rural areas where travel is difficult during winter months. Alternatively, rural patients may be less likely to undergo routine follow-up and presenting with advanced complications. Both of these potential explanations are consistent with our findings that rurality of patients’ residences is associated with a lower likelihood of diagnosis of many of the comorbidities.^33^ Residents of assisted living or nursing home facilities experienced twofold higher risk of mortality, but not PJI or revision surgeries. A plausible explanation for this finding is selection bias in which the frailest and those with highest disease burden transition from home to assisted living or nursing home facilities.

### Implications for Policy or Practice

Our findings indicate that neighborhood socioeconomic deprivation (ie, affordability and resources) is more strongly associated with TJA outcomes than geographic access to care. Further, the risk of death is higher in patients who underwent TJA living in institutionalized settings. Given these patterns, it is important to explore the pathways of patient experience in socioeconomically disadvantaged areas to identify targeted modifiable factors and reduce inequities in health care. Planned interventions should target these identified sources of disparities; for example, opportunities to improve or prevent decline in functional ADL skills would improve the risk of all 3 negative outcomes, whereas reducing obesity would reduce the risk of infections. Addressing many of the disparities in comorbidities would considerably improve the odds of averting adverse outcomes of TJA surgery.

These findings have implications for payment reform under the Comprehensive for Joint Replacement model, Medicare’s mandatory bundled payment model.^13^^,^^34^ Current incentives, focusing on outcomes, without addressing patient risk factors emerging from area deprivation would only serve to further enhance provider motivation to select patients with lower risk for negative outcomes based on geographic residence. This will only serve to exacerbate disparities in access to care for patients from neighborhoods associated with lower socioeconomic means. Findings can also inform decisions on regionalization of TJA care. It is well-established that high-volume centers have better short-term TJA outcomes than the low-volume centers. This study provides complementary evidence for long-term TJA outcomes on the basis of neighborhood-level socioeconomic deprivation and rurality.

### Limitations and Strengths

We were limited by lack of racial and ethnic minorities representation in our data, reducing generalizability of these findings to more racially and ethnically diverse geographic areas, and where factors other than area deprivation drive disparities. However, given our research question specifically examining the role of area-level measures of SES, our findings are not confounded with these factors of ethnicity and race, in fact providing a clearer look at the role of area-level deprivation on TJA outcomes. Second, our findings may not generalize to settings where rurality may have different implications for health care access than in the upper Midwestern United States. Third, we could not categorize education level further because educational attainment was collected differently over the years. Fourth, we did not examine readmissions, given our focus on long-term outcomes. Finally, to the extent that the publicly available census-based geocode system assigns the same area deprivation indices to all persons living in the census block group, we run the risk of misclassifying some individuals who do not experience the same level of deprivation because of factors such as transportation and other resources unaccounted for by this method of calculating individual-specific social determinants of health. Area deprivation index is a composite measure of neighborhood socioeconomic disadvantage and hence it is not possible to disentangle individual components of the ADI that include employment, income, poverty, and housing characteristics. Further, ADI and rurality is calculated based on 1 index year, the current norm for such calculations. We acknowledge some measurement error, as is typical of observation data, to the extent that ADI changes may have occurred in certain neighborhoods over the period studied. However, this index is considered stable across most geographic areas, especially as it relates to its impact on health. Finally, we examined only 3 measures of socioeconomic disadvantage. Other factors such as income, insurance status, or other area-level SES measures^29^^,^^35^ may be more relevant to TJA care than ADI, rurality, nursing home residency, and education. Income and insurance status are not routinely collected in this dataset. Despite these limitations, our analyses demonstrate the simultaneous and combined impact of several factors likely to contribute to outcomes. Given the recency of this stream of research, alternative methods should be compared with each other where possible to test the robustness of different neighborhood-level deprivation measures and postsurgical outcomes.^28^^,^^29^^,^^36^

One of the strengths of our study relates to the size and duration of follow-up of our cohort, making our findings robust and generalizable to similar settings, ie, large health systems serving a defined population over a relatively well-defined geographic area, with rich individual data on health over time, derived by linkages to their electronic medical records and geospatial information on their living environments. The size of the dataset also allowed us to look for interactions between area-based measures of deprivation and their impact in different patient groups. We were also able to compare the association of 2 separate area-based measures of deprivation on long-term surgical outcomes. Generalizability is a potential limitation because our cohort is restricted to patients who had surgery at a single, high-volume tertiary center, despite poor geographic access or socioeconomic disadvantages for some. However, restricting the study sample to a single center allowed us to avoid unmeasured confounding due to hospital-level factors, and demonstrate that performing TJA surgery at high-volume centers have potential to reduce socioeconomic disparities in TJA outcomes.

## Conclusion

Among patients who underwent TJA, neighborhood deprivation is most strongly associated with mortality but not with other surgical outcomes. Individual-level education moderates the impact of neighborhood deprivation on mortality. Patients living in areas with higher socioeconomic deprivation and in nursing homes are more likely to experience mortality, whereas only the highest level of socioeconomic deprivation and residency in rural small towns is associated with higher likelihood of revision surgery.

## Potential Competing Interests

Dr Berry has received royalties from DePuy, Johnson & Johnson Company, Elsevier, Wolters Kluwer Health and Lippincott Williams & Wilkins; received consulting fees from Bodycad, DePuy, and Johnson & Johnson Company; received honoraria from AO Recon; served on the board on “Current Concepts in Joint Replacement” (Hip Society and Knee Society), International Hip Society, Journal of Bone and Joint Surgery – American, and Orthopaedic Research and Education Foundation; share holdings in Bodycad; and received research from DePuy, and Johnson & Johnson Company. Dr Lewallen has received royalties on device related IP licensed by Department of Orthopedic Surgery, Mayo Clinic from Zimmer Biomet and Stryker; received consulting fees from Accuitive Technologies and Zimmer Biomet; served on the Advisory Board of Accuitive Technologies and Zimmer Biomet; is Managing Director of Mid America Orthopedic Association; committee member of Orthopedic Research and Education Foundation; and has share holdings of Accuitive Technologies and Ketai Medical Devices. All other authors declare that they have no competing interests.
